# Making the most of genomic data with OMA

**DOI:** 10.12688/f1000research.24904.1

**Published:** 2020-07-01

**Authors:** Natasha M. Glover

**Affiliations:** 1 Department of Computational Biology, University of Lausanne, Lausanne, 1015, Switzerland; 2 Swiss Institute of Bioinformatics, Lausanne, 1015, Switzerland; 3 Center for Integrative Genomics, Lausanne, 1015, Switzerland

**Keywords:** OMA, Orthologous Matrix, collection, orthologs

## Abstract

The OMA Collection is a resource for users of Orthologous Matrix. In this collection, we provide tutorials and protocols on how to leverage the tools provided by OMA to analyse your data. Here, I explain the motivation for this collection and its published works thus far.

Next generation sequencing has become commonplace, and we are now entering an age where whole genome sequences are a “dime a dozen.” Thousands of different eukaryotic species’ genomes have been sequenced to date, with certain species, such as humans, sequenced tens of thousands of times over. But this is just the tip of the iceberg! For example, the Earth BioGenome Project aims to sequence all 1.5 million known eukaryotic species in 10 years. The ‘-omics’ data presents layers of complexity in the form of gene expression, regulation, network interaction, epigenetics, structural, functional, and comparative genomics and more. With all this data comes a wealth of potential biological knowledge, but there must be efficient and smart ways to make sense out of all these genes and genomes.

One fundamental way to relate genomes is by orthology, or the relationship between genes of different species which originated from a single gene in the common ancestor of those species. It is commonly contrasted with paralogy, or the relationship between genes which originated by duplication. By tracing the evolutionary history of genes and their relationships between each other, we can start to understand the complexity of the biological processes underlying the evolution of life forms.

Indeed, there are many applications of orthology, such as:

- Prediction of gene function for uncharacterized proteins.
- Elucidating gene losses, duplications, or gains (i.e. taxonomically restricted genes) to study evolution of gene families and species.
- Finding the best model systems for study based on a particular physiological process.
- Phylogenetic profiling, or correlating ortholog presence or absence among many species to detect biologically related processes.
- Studying the positional conservation of genes, which can aid in genome assembly, homology detection, and provide insight into structural evolution of chromosomes.
- Phylogenomics, among others.

One particular tool for inferring orthologs is OMA (Orthologous MAtrix), which is a method and database for the inference of orthologs among complete genomes ^1^. Covering over 2000 species from a broad phylogenetic range, some distinctive features of the OMA browser include a feature-rich web interface, availability of data in a wide range of formats and interfaces, and twice yearly update schedule. As part of the Dessimoz lab, I have been working on the OMA project for the past 6 years, as well as being a user of the online browser and standalone software ^2^.

There are many orthology inference methods, software, and databases at one’s disposal. From working the past decade in computational biology, I have found that the bottlenecks for effectively using many bioinformatics tools are: 1) choosing the appropriate tool to fit your needs; 2) understanding the relevant information in the black box of how the tool works; and 3) efficiently running the tool and understanding the output.

Thus, the aim of this F1000 Research Collection is to provide a resource for users of OMA to help them with their analysis needs. I hope to make it as hassle-free as possible to use OMA and the many supplementary analysis tools currently provided. In this direction, we have written several Tutorials, Guides, and Protocols on how to use OMA to get the most out of one’s biological data.

So far, this collection contains four papers:

- Identifying orthologs with OMA: A primer ( *Software Tool article*) ^3^
- How to build phylogenetic species trees with OMA ( *Method article*) ^4^
- A hands-on introduction to querying evolutionary relationships across multiple data sources using SPARQL ( *Method article*) ^5^
- Expanding the Orthologous Matrix (OMA) programmatic interfaces: REST API and the OmaDB packages for R and Python ( *Software Tool article*) ^6^

All the aforementioned protocols use publicly available software, and we provide scripts, code snippets, practical examples, and plenty of explanations in order to facilitate the use of OMA in user analyses. We have several more tutorials planned for this collection and aim to continually add more resources to this collection in order to help our users.

Furthermore, with the goal of providing real-world examples on how OMA can be used, any research, commentaries, conference posters/slides, or other published work that uses OMA are welcome in this collection. Hopefully, the OMA Collection will prove to be a valuable resource for making the most of genomics data.

## Data availability

No data is associated with this article.
